# An oversampling-undersampling strategy for large-scale data linkage

**DOI:** 10.3389/fdata.2025.1542483

**Published:** 2025-04-23

**Authors:** Hossein Hassani, Mohammad Reza Entezarian, Sara Zaeimzadeh, Leila Marvian, Nadejda Komendantova

**Affiliations:** ^1^International Institute for Applied Systems Analysis (IIASA), Laxenburg, Austria; ^2^Department of Statistics, Shahid Beheshti University, Tehran, Iran; ^3^Department of Statistics, University of Tehran, Tehran, Iran; ^4^Big Data Lab, Imam Reza International University, Mashhad, Iran

**Keywords:** record linkage, data linkage, imbalanced datasets, oversampling, undersampling, big data

## Abstract

Effective record linkage in big data, particularly in imbalanced datasets, is a critical yet highly challenging task due to the inherent complexity involved. This article utilizes an oversampling-undersampling strategy to address linkage imbalances, enabling more accurate and efficient record linkage within large-scale datasets. It tries to increase the instances of the minority class and decrease the dominance of the majority classes to try to reach a more balanced dataset that can be used for training and testing. Sensitivity testing was carried out by varying the training-test ratio and degree of imbalance.

## 1 Introduction

Efficient record linkage is more than ever critical in big data analytics due to the large volume of information that mandates sound methodologies to ensure accurate identification and merging of records (Han et al., 2024a,b,c). Many applications operate on imbalanced datasets; this can compromise the efficiency and accuracy of various record linkage techniques (Hu et al., 2022; Cockburn et al., 2022). Imbalanced datasets represent the situation in which class distributions are not equal; thus, they produce unoptimized predictive performance, particularly for the minority class (Leevy et al., 2018; Chiang et al., 2024; Ayoub et al., 2023; Kraiem et al., 2021; Zou and Wang, 2024; Zhang et al., 2024; Gurcan and Soylu, 2024). This issue has been noted as a critical problem in many real-world applications such as fraud detection, medical diagnosis, marketing, finance, and anomaly detection as the target class is most often typically a minuscule fraction of all individuals (Bakator and Radosav, 2018; Mazurowski et al., 2008; Mahmudah et al., 2021; Amin et al., 2016; Sanz et al., 2015; Pang et al., 2021; de Zarzá et al., 2023). Also, most record linkage and data integration applications operate on imbalanced datasets (Du et al., 2021). While this is not a new problem, evidence from various applications shows that this continues to be a major challenge in various application areas (Johnson and Khoshgoftaar, 2019). Recently, a new R package, PreProcessRecordLinkage, was introduced, providing a robust framework for preprocessing data in record linkage tasks, highlighting its importance in ensuring seamless data integration and consistency across diverse datasets (Hassani and Mashhad, 2023). In this article, we present a methodology that combines both oversampling and undersampling techniques to tackle the imbalanced data issue in record linkage. The framework proposed in this study integrates both oversampling and undersampling strategies to enhance the efficiency and accuracy of record linkage. The synthetic over-representation of the minority classes and reduction of dominance in majority classes create a more balanced dataset for training and testing. Our proposed methodology is evaluated for sensitivity systematically, considering different sizes of training and test datasets under various degrees of data imbalance. The proposed methodology is experimented with a large volume dataset having varied attributes to prove applicability and usefulness in improving the accuracy and efficiency of record linkage in the context of big data analytics. Experiments indicate that our oversampling and undersampling approach can be used to address imbalanced data-related challenges, consequently leading to more sturdy and trustworthy results of record linkage.

The remainder of this article is structured as follows: in Section 2, we introduce the collection of classification models used in this study by revealing their mathematical grounding and applicability in the record linkage process. Section 2 also includes the performance evaluation measures and certain core metrics directly molding imbalanced datasets such as recall, precision, and F1-score.

Section 3 presents the proposed approach and methodology employed in this study. Section 4 denotes the experimental setup from which we draw our sources, attribute information, and preprocessing methods, along with a discussion on how blocking variables facilitate the simplification of pairwise comparisons.

Section 5 offers a detailed discussion of our results, the effect of different sampling strategies, and insights into model-specific performance. Section 6 provides a concise overview of our primary findings, explores potential real-world applications, and suggests avenues for further refining record linkage approaches in big data contexts.

## 2 Methods and criteria

### 2.1 Methods

This section details our approach employed in this study. We applied multiple classification models to the dataset, aiming to identify the most effective strategy under imbalanced conditions. Here, the following models were selected:

**Notation:**

- *Y*: Binary outcome taking the value 1 for a match and 0 for a nonmatch.
- *X* = (*X*_1_, *X*_2_, …, *X*_*p*_): Vector of record pair characteristics.
- β_0_, β_1_, …, β_*p*_: Parameters estimated from the model.
- *P*(*Y* = 1|*X*): Probability that the pair of records is a match given features *X*.
- *k*: Number of nearest neighbors.
- *T*_*b*_(*X*): Prediction from the *b*-th tree in the ensemble.
- *h*_*m*_(*X*): Prediction from the *m*-th weak learner in AdaBoost.^1^
- α_*m*_: The weight of the *m*-th weak learner.
- *f*_*m*_(*X*): Prediction from the *m*-th tree in ensemble-based boosting models.

1. **Logistic regression (LR):** Logistic Regression models the probability *P*(*Y* = 1|*X*) that a record pair is a match given feature vector *X*. This is defined as:

$$P\left(Y=1|X\right)=\frac{1}{1+e^{-\left(\beta_{0}+\beta_{1}X_{1}+\cdots +\beta_{p}X_{p}\right)}}$$

where β_0_, β_1_, …, β_*p*_ are the parameters learned from the data. This method is particularly useful for binary classification tasks like record linkage.
2. **Naïve Bayes (NB):** Naïve Bayes leverages Bayes' theorem to calculate the posterior probability of each class given the features. Assuming feature independence, the probability of a record pair being a match is:

$$P\left(Y=1|X\right)=\frac{P\left(Y=1\right)\prod_{i=1}^{p}P\left(X_{i}|Y=1\right)}{\sum_{y\in \left\{0,1\right\}}P\left(Y=y\right)\prod_{i=1}^{p}P\left(X_{i}|Y=y\right)}$$

where *P*(*Y* = 1) is the prior probability of a match, and *P*(*X*_*i*_|*Y*) are the conditional probabilities of each feature given the class.
3. **K-nearest neighbors (KNN):** KNN classifies a record pair by examining the classes of its *k*-nearest neighbors in feature space. The predicted class *Ŷ* for a record pair *x* is determined by majority vote:

$$\hat{Y}=\text{mode}\left\{Y_{\left(1\right)},Y_{\left(2\right)},\ldots ,Y_{\left(k\right)}\right\}$$

where *Y*_(*i*)_ is the class label of the *i*-th nearest neighbor to *x*.
4. **Random forest (RF):** Random Forest constructs an ensemble of decision trees {*T*_1_(*X*), *T*_2_(*X*), …, *T*_*B*_(*X*)} and averages their predictions to classify a record pair. For classification, the final prediction is:

$$\hat{Y}=\text{mode}\left(T_{1}\left(X\right),T_{2}\left(X\right),\ldots ,T_{B}\left(X\right)\right)$$

where each *T*_*b*_(*X*) is a tree trained on a bootstrapped sample of the data.
5. **Decision tree (DT):** A Decision Tree splits the feature space recursively to maximize information gain at each split, ultimately assigning a class to each leaf node. The prediction for a new record pair *X* is given by traversing the tree based on feature values until reaching a leaf with the assigned class Ŷ.
6. **AdaBoost:** AdaBoost sequentially builds an ensemble by adjusting weights to focus on previously misclassified instances. The final model prediction is:

$$\hat{Y}=\text{sign}\left(\overset{M}{\underset{m=1}{\sum }}\alpha_{m}h_{m}\left(X\right)\right)$$

where *h*_*m*_(*X*) is the prediction of the *m*-th weak learner, and α_*m*_ is the weight given to each learner based on its accuracy.
7. **XGBoost (extreme gradient boosting)**^2^: XGBoost builds an ensemble of trees by optimizing a regularized objective. The prediction for record pair *X* is:

$$\hat{Y}=\overset{M}{\underset{m=1}{\sum }}f_{m}\left(X\right)$$

where *f*_*m*_(*X*) represents the *m*-th tree, and each tree is optimized to minimize the loss function with regularization to reduce overfitting.
8. **Gradient boosting:** Gradient Boosting builds a model iteratively by adding weak learners to minimize the residual error. The prediction for *X* is:

$$\hat{Y}=\overset{M}{\underset{m=1}{\sum }}f_{m}\left(X\right)$$

where *f*_*m*_(*X*) represents the *m*-th weak learner, typically a decision tree, added to correct errors made by the previous learners.

Central to the modeling approach was the dataset's imbalanced nature. Given the all-encompassing combinations of the two sets, there's a significant skew toward non-links. To ameliorate this, two renowned strategies were adopted:

1. Oversampling: Augmenting the minority class representation by duplicating or synthesizing data.
2. Undersampling: Curtailing the majority class instances to achieve class balance.

### 2.2 Criteria

For binary classification problems, understanding and utilizing a confusion matrix as well as critical metrics is key to a thorough assessment. It provides an explicit outline of how classification outcomes can be measured to thoroughly evaluate the model's performance based on metrics like true-positives, true-negatives, false-positives, and false-negatives (see Table 1). A more detailed and informative analysis of the model's strengths and weaknesses allows for a better understanding of model performance in binary classification scenarios. While data imbalances can potentially make accuracy a dubious metric, we were mostly interested in the financial fallout of false negatives where real links would have been misclassified as non-links. Hence, Recall was chosen as our prime evaluation metric to assess how well the model would identify such relevant cases.


$$\text{Recall}=\frac{TP}{TP+FN}$$

**Table 1:** Confusion matrix.

	**Predicted unpair**	**Predicted pair**
Actual unpair	True negative (TN)	False positive (FP)
Actual pair	False negative (FN)	True positive (TP)

Though, the use of measures such as Accuracy, Precision, and the F1 Score greatly improves the trustworthiness and comprehensiveness of the evaluation techniques. It focuses on Accuracy; therefore, it gives the correct picture of the general effectiveness of a model by focusing on the correct classification of instances. Focusing on Precision helps cut down false positives, giving an acute understanding of how well the model can make positive predictions without being incorrect. The F1 Score gives a measure that combines both Precision and Recall into one single measure and, in such a way, helps balance this tradeoff between the two. It is especially designed to be more dataset balanced, unlike the Accuracy. All these metrics being introduced in academic work readings and reviews do more than just improving the review or assessment of machine learning models –it strengthens and deepens the whole analytical structure.


$$\text{Precision}=\frac{\text{True Positives}}{\text{True Positives }+\text{ False Positives}}$$

$$F1=2\times \frac{\text{Precision }\times \text{ Recall}}{\text{Precision }+\text{ Recall}}$$

## 3 Proposed approach

The core motivation behind our proposed approach is to tackle the highly imbalanced nature of record linkage datasets through a strategy that considers both oversampling and undersampling. By striking a more equitable class distribution, we aim to improve each classifier's ability to detect matches (the minority class) without sacrificing performance on the non-match majority class.

### 3.1 Oversampling and undersampling strategy

Two complementary sampling techniques–oversampling and undersampling–form the basis of our proposed method:

1. Oversampling: This increases the representation of the minority (match) class. We specifically employ variants of Synthetic Minority Oversampling TEchnique (SMOTE) and ROSE to generate synthetic data points that reflect the underlying distribution of real matches. Unlike naive duplication, synthetic oversampling helps the model generalize better by introducing slightly varied examples rather than simple repeats.
2. Undersampling: This reduces the abundance of the majority (non-match) class, thus forcing the classifier to learn nuanced decision boundaries. A smaller set of carefully retained majority examples helps models avoid bias toward the dominant class. This is particularly useful where the total number of non-match records significantly outweighs the matches.

In our pipeline, we explore three main variations for each dataset:

- *Oversampled:* Synthetic generation of additional match instances (e.g., via SMOTE or ROSE) while leaving the majority class intact.
- *Undersampled:* Random removal (or more sophisticated selection) of non-match instances to reduce the non-match pool to a level comparable to the match class.

Depending on the degree of imbalance and dataset size, any of these two approaches can be employed.

### 3.2 Evaluation protocol

To rigorously assess the effectiveness of the proposed approach, we rely on a set of standard metrics–Accuracy, Precision, Recall, and F1 Score–and pay special attention to Recall in light of the high cost of false negatives in record linkage. We apply multiple train-test splits (e.g., 70/30, 60/40) and artificially remove fixed quantities of matches (e.g., 500, 1000) to simulate worst-case imbalances. Each experiment is repeated for:

- *Original data*: No rebalancing performed.
- *Oversampled/undersampled data*: Rebalanced via one of the methods above.

For each split and each sampling method, all eight classification models are trained and evaluated. By comparing performance metrics under these different conditions, we gain insight into the generalizability of our approach.

### 3.3 Advantages of the proposed approach

1. Improved minority detection: the sampling strategy proposed here aids in capturing subtle boundary distinctions that might otherwise be lost due to extreme imbalance.
2. Flexibility: depending on the severity of imbalance, a purely oversampled or purely undersampled option can be used, or both methods can be combined for maximum effect.
3. Algorithm-Agnostic: our sampling enhancements are pre-processing steps, meaning they can be applied seamlessly to a wide array of classification algorithms without needing custom changes.
4. Robustness to reduced matches: even when matches are removed (simulating missing or hard-to-collect labels), the synthetic minority examples help preserve recall and stabilize F1 scores.

The sampling framework presented here, coupled with our evaluation scheme, constitutes the crux of our proposed method. We show in subsequent sections that this approach consistently boosts recall and F1, making it a suitable choice for challenging record linkage tasks with highly imbalanced data.

## 4 Data and pre-processing

### 4.1 General information

The data set utilized here called the Matching German Epidemiological Cancer Study and compiled by extracting relevant records from a research database.^3^ More precisely, the database formation falls under a wider Cancer Epidemiology Study database carried out conjointly by the Institute of Medical Biometrics, Epidemiology and Computer Science (IMBEI) and University Medical Center of Johannes Gutenberg University, Mainz, Germany. It includes individualized details with first and last names, sex, date of birth, and postal code; these have been collected continually over the past few years. The file used for matching is specifically derived from a subset of 100,000 records collected between 2005 and 2008. Each pair of data was subsequently processed through a rather extensive manual review process that involved several documentalists, who finally arrived at a classification of it being a “match” or “non-match.” This manual classification therefore serves as the gold standard against which to measure the performance and accuracy of the registry's in-house record linkage methodology.

### 4.2 Blocking variables

To simplify the pattern analysis and decrease the volume, a blocking procedure is used, which selectively isolates those record pairs that meet certain predefined agreement conditions. The results of six different blocking iterations were then combined:

1. Phonetic congruence of both the first and family names, coupled with an exact match of the date of birth.
2. Phonetic congruence of the first name, with an exact match of the birthday.
3. Phonetic congruence of the first name, with an exact match of the birth month.
4. Phonetic congruence of the first name, with an exact match of the birth year.
5. An exact match of the entire date of birth.
6. Phonetic congruence of the family name, along with an exact match of sex.

Upon execution of this procedure, a total of 5,749,132 record pairs were generated. Of these, 20,931 were identified as matches. It does so by dividing the dataset into 10 blocks of approximately equal size; within each block, the number of non-matches for every match is the same as in any other block.

### 4.3 Attribute information

Attributes are the characteristics of data records. In other words, for each individual in your dataset, it has several features that help in differentiating or understanding specific aspects of the record. The set of attributes you provided seems to belong to a dataset whose use is for the sake of comparing or matching. For some of these attributes, when specified to be in the range [0, 1], it qualifies agreement to mean that 0 indicates fully dissimilar for that attribute, and 1 requires identical.

1. **id_1**: internal identifier of first record.
2. **id_2**: internal identifier of second record.
3. **cmp_fname_c1**: agreement of first name, first component.
4. **cmp_fname_c2**: agreement of first name, second component.
5. **cmp_lname_c1**: agreement of family name, first component.
6. **cmp_lname_c2**: agreement of family name, second component.
7. **cmp_sex**: agreement of sex.
8. **cmp_bd**: agreement of date of birth, day component.
9. **cmp_bm**: agreement of date of birth, month component.
10. **cmp_by**: agreement of date of birth, year component.
11. **cmp_plz**: agreement of postal code.
12. **is_match**: matching status (TRUE for matches, FALSE for non-matches).

Let us now briefly describe the attribute mentioned above.

**id_1 & id_2**: These are likely unique identifiers assigned to each record in the dataset. These IDs help in recognizing and referencing each individual record without confusion.

**cmp_fname_c1 & cmp_fname_c2**: These attributes are used to measure the agreement or similarity between the first components and second components of first names, respectively, in the compared records.

**cmp_lname_c1 & cmp_lname_c2**: Similarly, these measure the agreement or similarity between the first and second components of family names in the compared records.

**cmp_sex**: This attribute gauges the agreement on sex between the compared records.

**cmp_bd, cmp_bm, & cmp_by**: These denote the level of agreement between the day, month, and year components of the date of birth of compared records.

**cmp_plz**: Represents the agreement between postal codes of the compared records.

**is_match**: A Boolean attribute that indicates whether the two records being compared are a match or not.

### 4.4 Missing values

The table provided below illustrates the tally of missing data for each attribute. The subsequent table details the completeness of the data across attributes in a dataset. Noteworthy among these are the unique identifiers, *id*1 and *id*2, cmplsex and Linkage, all of which have no missing entries. In the meantime, *cmp*_*f*_*irstname*2 and *cmp*_*l*_*astname*2 have by far the most missing data, where 5,645,434 and 5,746,668 records are not available, respectively. Also, the observed variations regarding agreement on date of birth components for *cmp*_*b*_*irthday*, *cmp*_*b*_*irthmonth*, and *cmp*_*b*_*irthyear* find 795 values missing in each case, whereas *cmp*_*z*_*ipcode* has 12,843 missing entries. Table 2 is important for understanding dataset quality throughout data preprocessing and analysis to make sound decisions.

**Table 2:** Summary of missing values for each attribute.

**Attribute**	**Missing values**
id1	0
id2	0
cmp_firstname1	1,007
cmp_firstname2	5,645,434
cmp_lastname1	0
cmp_lastname2	5,746,668
cmp_sex	0
cmp_birthday	795
cmp_birthmonth	795
cmp_birthyear	795
cmp_zipcode	12,843
Linkage	0

Given that more than 98% of entries in the *cmp*_*firstname*2 and *cmp*_*lastname*2 columns are missing, including these variables in the analysis would contribute little to the overall information content. Moreover, retaining them would require extensive imputation or data-cleaning steps, potentially skewing the results. Consequently, these two columns have been entirely omitted, allowing the analysis to focus on attributes with sufficient data coverage and minimizing sources of bias.

## 5 Results

### 5.1 General overview

To gain a deeper insight into how varying levels of imbalance affect recall, we comprehensively evaluated the models under three distinct scenarios:

1. **Original data**: an untouched reflection of the dataset.
2. **After excluding 5,000 linked rows**: a dataset variant post the strategic removal of 5,000 linked records.
3. **After excluding 10,000 linked rows**: another iteration post the omission of 10,000 linked records.

These will give conclusive information about generalization capabilities, flexibility, and efficiency for each model in different data setups. Reporting the variation in recall performance across datasets, for the different algorithms, is shown in Table 3. The datasets were drawn from different sampling strategies as indicated by Original Data, Oversampled Data, and Undersampled Data. For each such dataset, recall performance was calculated with different test set proportions. It considered 10%, 20%, 30%, and 40%.

**Table 3:** Performance metrics of classification models on imbalanced datasets using various sampling techniques.

**Test: 10%**									
		**LR**	**NB**	**KNN**	**RF**	**DT**	**AdaB**	**XGB**	**GRB**
Original	Recal	99.71%	95.26%	99.90%	99.85%	99.85%	99.85%	99.85%	99.61%
Oversample(SMOTE)		100%	95.26%	100%	99.85%	99.90%	99.90%	99.90%	99.90%
Oversampled(Rose)		100%	99.90%	99.95%	99.83%	99.80%	100.00%	99.95%	100.00%
Undersample		100%	99.90%	100%	100.00%	99.95%	100.00%	100.00%	100.00%
Original	Accuracy	99.99%	99.98%	99.99%	99.99%	99.99%	99.99%	99.99%	99.99%
Oversample(SMOTE)		99.97%	99.98%	99.99%	99.99%	99.99%	99.99%	99.99%	99.99%
Oversampled(Rose)		99.97%	98.56%	99.99%	99.65%	99.99%	99.99%	99.99%	99.98%
Undersample		99.98%	98.56%	99.96%	100.00%	99.94%	99.98%	99.97%	99.98%
Original	Precision	99.71%	98.60%	99.61%	99.87%	99.75%	99.85%	99.90%	99.46%
Oversample(SMOTE)		94.69%	98.60%	97.50%	99.85%	99.66%	99.47%	99.75%	99.27%
Oversampled(Rose)		94.69%	19.98%	98.61%	99.78%	99.85%	98.10%	99.75%	96.90%
Undersample		95.21%	19.98%	91.10%	100.00%	86.45%	94.82%	93.45%	95.03%
Original	F1	99.71%	96.90%	99.76%	99.86%	99.81%	99.85%	99.87%	99.54%
Oversample(SMOTE)		97.27%	96.90%	98.73%	99.85%	99.78%	99.68%	99.83%	99.59%
Oversampled(Rose)		97.27%	33.30%	99.27%	99.78%	99.83%	99.04%	99.85%	96.90%
Undersample		97.55%	33.30%	95.34%	100.00%	92.71%	97.34%	96.61%	97.45%
**Test: 20%**									
		**LR**	**NB**	**KNN**	**RF**	**DT**	**AdaB**	**XGB**	**GRB**
Original	Recall	99.65%	95.22%	99.51%	99.85%	99.87%	99.90%	99.87%	99.60%
Oversample(SMOTE)		100%	95.22%	99.95%	99.85%	99.90%	99.90%	99.92%	99.85%
Oversampled(Rose)		100%	99.78%	99.95%	99.92%	99.82%	99.97%	99.92%	100.00%
Undersample		100%	99.77%	99.97%	100.00%	100.00%	100.00%	100.00%	100.00%
Original	Accuracy	99.99%	99.98%	99.99%	99.99%	99.99%	99.99%	99.99%	99.99%
Oversample(SMOTE)		99.98%	99.98%	99.99%	99.99%	99.99%	99.99%	99.99%	99.99%
Oversampled(Rose)		99.98%	98.54%	99.99%	99.92%	99.99%	99.99%	99.99%	99.98%
Undersample		99.98%	98.54%	99.96%	100.00%	99.92%	99.97%	99.96%	99.97%
Original	Precision	99.77%	98.65%	99.80%	99.87%	99.75%	99.87%	99.87%	99.43%
Oversample(SMOTE)		96.77%	98.65%	97.63%	99.85%	99.65%	99.31%	99.73%	99.17%
Oversampled(Rose)		94.71%	19.61%	97.79%	99.92%	99.75%	97.96%	99.75%	96.73%
Undersample		94.91%	19.61%	91.01%	100.00%	83.07%	92.58%	91.81%	92.60%
Original	F1	99.71%	96.91%	99.65%	99.86%	99.82%	99.88%	99.87%	99.52%
Oversample(SMOTE)		98.36%	96.91%	98.77%	99.85%	99.77%	99.60%	99.82%	99.51%
Oversampled(Rose)		97.28%	32.78%	98.86%	99.92%	99.79%	98.95%	99.84%	98.33%
Undersample		97.38%	32.78%	95.28%	100.00%	90.75%	96.15%	95.73%	96.16%
**Test: 30%**									
		**LR**	**NB**	**KNN**	**RF**	**DT**	**AdaB**	**XGB**	**GRB**
Original	Recall	99.61%	95.04%	99.25%	99.75%	99.82%	99.80%	99.74%	99.54%
Oversample(SMOTE)		99.98%	95.04%	99.96%	99.88%	99.86%	99.90%	99.90%	99.78%
Oversampled(Rose)		100%	99.74%	99.96%	99.92%	99.82%	99.98%	99.93%	99.98%
Undersample		99.96%	99.74%	99.93%	100.00%	100.00%	99.98%	100.00%	99.98%
Original	Accuracy	99.99%	99.97%	99.99%	99.99%	99.99%	99.99%	99.99%	99.99%
Oversample(SMOTE)		99.98%	99.98%	99.99%	99.99%	99.99%	99.99%	99.99%	99.99%
Oversampled(Rose)		99.97%	98.53%	99.99%	99.99%	99.99%	99.99%	99.99%	99.98%
Undersample		99.98%	98.54%	99.98%	99.98%	99.94%	99.98%	99.97%	99.98%
Original	Precision	99.82%	98.85%	99.90%	99.93%	99.77%	99.90%	99.88%	99.51%
Oversample(SMOTE)		96.89%	99.97%	98.10%	99.98%	99.90%	99.50%	99.96%	99.45%
Oversampled(Rose)		94.13%	19.70%	98.26%	99.51%	99.96%	98.31%	99.91%	97.10%
Undersample		96.07%	19.71%	95.30%	96.08%	87.16%	94.79%	94.29%	95.47%
Original	F1	99.71%	96.91%	99.57%	99.84%	99.80%	99.85%	99.81%	99.53%
Oversample(SMOTE)		98.41%	96.91%	99.02%	99.93%	99.89%	99.70%	99.93%	99.62%
Oversampled(Rose)		96.97%	32.91%	99.10%	99.93%	99.89%	99.14%	99.92%	98.52%
Undersample		97.85%	32.91%	97.56%	98.00%	93.14%	97.32%	97.06%	97.67%
**Test: 40%**									
		**LR**	**NB**	**KNN**	**RF**	**DT**	**AdaB**	**XGB**	**GRB**
Original	Recall	94.95%	95.04%	99.44%	99.72%	99.80%	99.77%	99.73%	99.58%
Oversample(SMOTE)		99.97%	95.04%	99.87%	99.78%	99.80%	99.85%	99.85%	99.77%
Oversampled(Rose)		99.98%	99.70%	99.86%	99.75%	99.75%	99.93%	99.89%	99.97%
Undersample		99.98%	99.69%	99.92%	99.98%	99.97%	99.98%	99.97%	99.97%
Original	Accuracy	99.96%	99.97%	99.99%	99.99%	99.99%	99.99%	99.99%	99.99%
Oversample(SMOTE)		99.98%	99.97%	99.98%	99.99%	99.99%	99.99%	99.99%	99.99%
Oversampled(Rose)		99.97%	98.54%	99.98%	99.99%	99.99%	99.99%	99.99%	99.98%
Undersample		99.98%	98.54%	99.97%	99.98%	99.93%	99.97%	99.99%	99.98%
Original	Precision	96.42%	98.82%	99.67%	99.91%	99.83%	99.91%	99.90%	98.29%
Oversample(SMOTE)		96.88%	98.82%	96.64%	99.90%	99.74%	99.43%	99.79%	99.24%
Oversampled(Rose)		94.73%	19.76%	96.64%	99.93%	99.88%	98.34%	99.84%	97.28%
Undersample		95.72%	19.76%	94.25%	99.74%	85.12%	93.95%	93.20%	95.17%
Original	F1	95.68%	96.89%	99.55%	99.82%	99.82%	99.84%	99.81%	98.93%
Oversample(SMOTE)		98.40%	96.89%	98.23%	99.84%	99.77%	99.64%	99.82%	99.50%
Oversampled(Rose)		97.28%	32.98%	98.23%	99.84%	99.81%	99.13%	99.86%	98.61%
Undersample		97.81%	32.98%	97.00%	97.82%	91.95%	96.87%	96.47%	97.51%

**Figure 1:**
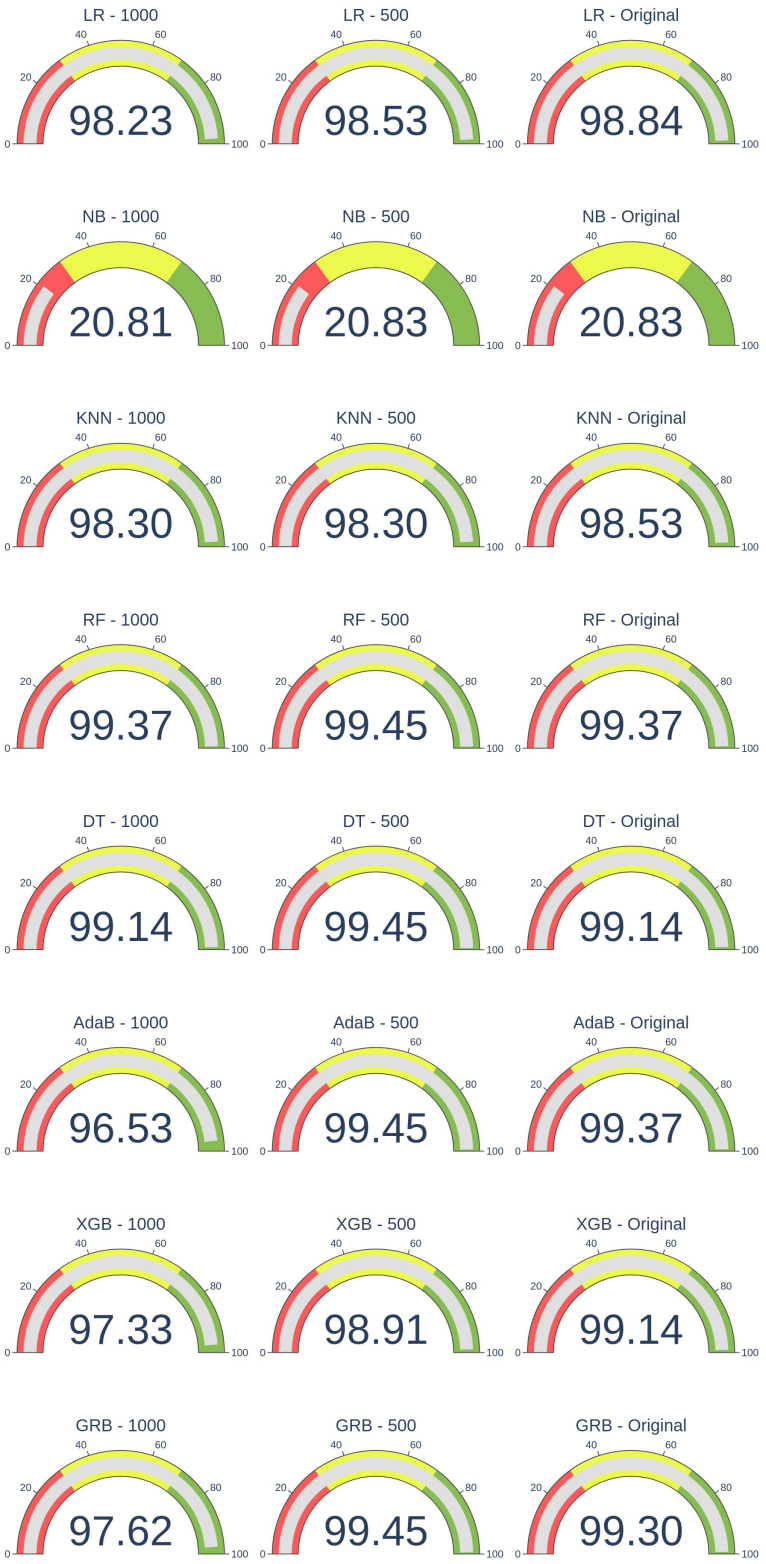
Effect of SMOTE on F1-scores under different levels of data imbalance.

The gauge plots (Figure 1) highlight the effectiveness of the SMOTE technique in maintaining consistent F1-scores across various scenarios in an imbalanced record linkage task. Even after changing the number of matches, the F1-scores remain stable for all models, demonstrating SMOTE's ability to mitigate the effects of imbalance and preserve classification performance. This consistency underscores SMOTE's robustness in addressing data imbalance, ensuring reliable results even when the number of matches is reduced.

**Figure 2:**
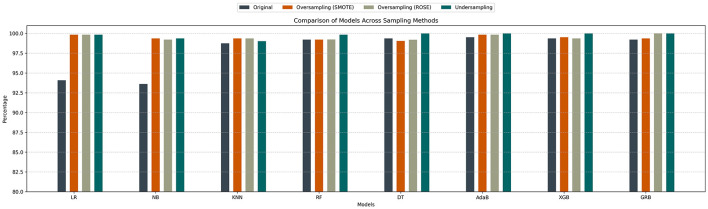
Comparison of sampling methods for recall improvement under worst-case imbalance.

The bar chart (Figure 2) illustrates the effectiveness of different sampling methods–SMOTE, ROSE, and undersampling–in improving recall scores under a worst-case scenario of 1000 matches removed within a 70/30 train-test split. Across all eight machine learning models, these sampling techniques consistently increase recall compared to the original dataset, demonstrating their ability to address class imbalance. While the extent of improvement varies between models, the overall results underscore the value of both oversampling and undersampling approaches in mitigating imbalance and boosting performance in difficult conditions.

### 5.2 Detailed breakdown of results

Below is a metric-by-metric discussion of the table's outcomes, highlighting observations for each sampling method (Original, SMOTE, ROSE, Undersample), classifier (LR, NB, KNN, RF, DT, AdaB, XGB, GRB), and test split (10%, 20%, 30%, 40%).

#### 5.2.1 Recall

- **Original data:** Most ensemble models (RF, DT, AdaB, XGB, GRB) achieve very high recall (close to or above 99%). Naïve Bayes (NB) hovers around 95%, while Logistic Regression (LR) ranges between roughly 95%–99.7% depending on the test split.
- **Oversample (SMOTE):** Raises recall values to near 100% for almost all models, illustrating SMOTE's ability to bolster the minority class. KNN sees slight improvements, whereas other models hit or approach 100% at smaller test splits.
- **Oversampled (ROSE):** Often comparable to SMOTE, pushing many models (especially ensembles) to near 99-100%. However, a few models (e.g., NB, KNN) can vary more with the test split.
- **Undersample:** Produces near-100% recall across nearly all models and test sizes, indicating very effective minority-class detection.

#### 5.2.2 Accuracy

- **Minimal variations:** Overall accuracy remains consistently around 99.9% for nearly every model-sampling combination. This indicates that the large majority class is rarely misclassified in substantial numbers.

#### 5.2.3 Precision

- **Original data:** Typically high across the board (98–99%), especially for ensemble methods and LR. NB also remains in the high-90% range.
- **Oversample (SMOTE):** In some cases, SMOTE slightly reduces precision for LR or KNN (a small increase in false positives). Most models, however, remain at 95%–98% precision or higher.
- **Oversampled (ROSE):** Similar to SMOTE but can cause more variability. Some models see improved recall at the expense of precision (especially LR or NB), whereas robust ensemble methods often keep precision above 99%.
- **Undersample:** Precision dips for some algorithms (e.g., NB) due to fewer majority samples. Yet, ensemble models typically maintain around 99%.

#### 5.2.4 F1 score

- **Original data:** Already very strong (often 95–99%), especially for ensemble methods, which exceed 99% in many splits. NB and LR are slightly lower but still in the mid- to high-90s.
- **Oversample (SMOTE):** Boosts F1 for models needing help on the minority class (e.g., LR, KNN), pushing them closer to 98–99%. Ensemble classifiers remain near or above 99%.
- **Oversampled (ROSE):** Some scenarios match SMOTE's effectiveness, though results occasionally fluctuate more. Gains in recall might come at a small cost to precision, leading to variable F1 across test splits.
- **Undersample:** Often leads to high F1, given the near-perfect recall. A moderate dip in precision for some models can slightly lower F1, but most scores remain well above 90%.

#### 5.2.5 Effect of test split (10%, 20%, 30%, 40%)

Across all sampling methods, varying the test split has minimal impact on the overall ranking of model performance. Ensemble classifiers (RF, DT, AdaB, XGB, GRB) consistently top both recall and F1 scores. NB and LR, which start with lower recall or precision, see bigger gains from oversampling or undersampling.

### 5.3 More pronounced effect of over- and under-sampling

To assess the impact of different resampling techniques on model performance, we evaluate over-sampling (SMOTE and ROSE) and under-sampling approaches using a subset of the original dataset. This subset is selected to illustrate the extent to which sampling strategies influence model recall. Table 4 summarizes the recall improvement across various machine learning models when different resampling techniques are applied.

**Table 4:** The recall improvement of different resampling techniques.

**Model**	**Original**	**Smote (%)**	**Rose (%)**	**Under-sample (%)**
LR	85.25	98.80 (15.89%)	99.00 (16.13%)	88.80 (4.16%)
NB	98.80	99.95 (1.16%)	99.95 (1.16%)	100.00 (1.21%)
KNN	84.10	97.04 (15.39%)	89.40 (6.30%)	99.10 (17.84%)
RF	87.15	99.75 (14.46%)	99.37 (14.02%)	99.37 (14.02%)
DT	89.90	97.25 (8.18%)	96.70 (7.56%)	98.10 (9.12%)
AdaB	95.75	96.60 (0.89%)	96.60 (0.89%)	97.50 (1.83%)
XGB	89.90	97.45 (8.40%)	97.45 (8.40%)	89.90 (0.00%)
GRB	87.60	99.10 (13.13%)	98.10 (11.99%)	99.90 (14.04%)

The results highlight a notable improvement in recall for most models when over-sampling techniques such as SMOTE and ROSE are applied. For example, Logistic Regression sees a 16.13% improvement with ROSE and 15.89% with SMOTE compared to the original dataset.

Interestingly, under-sampling also yields performance gains, particularly in models like KNN (17.84%) and GRB (14.04%), indicating that reducing the majority class can sometimes enhance model generalization.

These findings emphasize the importance of selecting appropriate sampling techniques based on the model type and dataset characteristics. Over-sampling methods generally provide consistent recall improvements, while under-sampling can be beneficial in some cases but may lead to information loss, potentially reducing generalization for certain models.

## 6 Conclusion

This study confirms the critical role of accurate record linkage, especially when dealing with complex cancer-related datasets. While manual review has traditionally been the mainstay of linkage procedures, our findings show that modern machine learning classifiers offer consistently high recall and F1 scores, underscoring their potential for improving linkage accuracy and efficiency.

Furthermore, the results highlight that the choice of sampling strategy–oversampling (e.g., SMOTE or ROSE) or undersampling–significantly influences a model's ability to identify minority (match) cases. In many scenarios, these strategies boosted recall to near-perfect levels, thereby reducing the risk of missing critical matches in large, imbalanced datasets. Moving forward, leveraging advanced machine learning algorithms in conjunction with carefully selected sampling methods stands to greatly enhance record linkage outcomes, ultimately paving the way for more robust and insightful epidemiological investigations.

Creating a dedicated R package, similar to the approach taken in Hassani and Mashhad (2023) for preprocessing in record linkage, would be a valuable addition for this task. Such a package could simplify the implementation process and deliver actionable results to researchers and practitioners who may not be familiar with all the methods used, thereby increasing accessibility and efficiency in record linkage practices.

## Data Availability

Publicly available datasets were analyzed in this study. This data can be found here: based on request.

## Footnotes

^1^AdaBoost (Adaptive Boosting) was originally introduced by Yoav Freund and Robert E. Schapire in 1995. In this study, we utilized the implementation available in the Python-based scikit-learn library (version 1.2.2), which provides a robust and widely adopted framework for machine learning applications.

^2^XGBoost (Extreme Gradient Boosting) was developed by Tianqi Chen and Carlos Guestrin and is publicly available as an open-source library on GitHub. The implementation used in this study is the Python package xgboost (version 1.7.4), known for its high performance, scalability, and accuracy in supervised learning tasks.

^3^Available online at: https://archive.ics.uci.edu/dataset/210/record+linkage+comparison+patterns
